# Sperm Competition Selects for Sperm Quantity and Quality in the Australian Maluridae

**DOI:** 10.1371/journal.pone.0015720

**Published:** 2011-01-25

**Authors:** Melissah Rowe, Stephen Pruett-Jones

**Affiliations:** 1 Department of Ecology and Evolution, University of Chicago, Chicago, Illinois, United States of America; 2 National Centre for Biosystematics, Natural History Museum, University of Oslo, Oslo, Norway; Roehampton University, United Kingdom

## Abstract

When ejaculates from rival males compete for fertilization, there is strong selection for sperm traits that enhance fertilization success. Sperm quantity is one such trait, and numerous studies have demonstrated a positive association between sperm competition and both testes size and the number of sperm available for copulations. Sperm competition is also thought to favor increases in sperm quality and changes in testicular morphology that lead to increased sperm production. However, in contrast to sperm quantity, these hypotheses have received considerably less empirical support and remain somewhat controversial. In a comparative study using the Australian Maluridae (fairy-wrens, emu-wrens, grasswrens), we tested whether increasing levels of sperm competition were associated with increases in both sperm quantity and quality, as well as an increase in the relative amount of seminiferous tubule tissue contained within the testes. After controlling for phylogeny, we found positive associations between sperm competition and sperm numbers, both in sperm reserves and in ejaculate samples. Additionally, as sperm competition level increased, the proportion of testicular spermatogenic tissue also increased, suggesting that sperm competition selects for greater sperm production per unit of testicular tissue. Finally, we also found that sperm competition level was positively associated with multiple sperm quality traits, including the proportion of motile sperm in ejaculates and the proportion of both viable and morphologically normal sperm in sperm reserves. These results suggest multiple ejaculate traits, as well as aspects of testicular morphology, have evolved in response to sperm competition in the Australian Maluridae. Furthermore, our findings emphasize the importance of post-copulatory sexual selection as an evolutionary force shaping macroevolutionary differences in sperm phenotype.

## Introduction

When females copulate with multiple males during a single reproductive episode, sperm from these males compete to fertilize the female's ova in a process known as sperm competition [1]. Sperm competition is a powerful selective force that favours male traits that maximize competitive fertilization success. Across a diverse range of taxa, comparative and experimental studies have demonstrated that a common evolutionary response to sperm competition is an increase in testes size [2]–[7], [ see also 8]. Indeed, relative testis size is often used as a measure of sperm competition [e.g. 9]–[12]. Furthermore, inter- and intra-specific studies suggest that sperm competition is positively associated with greater numbers of sperm (i.e. sperm reserves or ejaculate size) [5], [13]–[15]. This is at least partially because larger testes produce more sperm [16]–[20]. However, in addition to testes size, sperm competition may select for increases in sperm production: species under higher sperm competition have a greater proportion of sperm-producing tissue within the testes [21], [22]. Currently, however, there is limited empirical data concerning testis morphology and additional studies are clearly warranted in order to more fully understand the links between sperm numbers, sperm production and sperm competition.

Sperm competition is also thought to favor a range of sperm phenotypic traits that influence the fertilizing capability of an ejaculate [23], [24]. For example, sperm motility influences paternity success in a range of taxa (e.g. birds [25]–[27], fish [28], [29], mammals [30]) and, across species, there is a positive association between the intensity of sperm competition and sperm swimming speed (birds [31], [ but see 32], fish [33]). Additionally, sperm competition is associated with changes in sperm design (e.g. morphology) and function (e.g. sperm energetics) that influence swimming velocity [34]–[36]. More generally, sperm competition appears to be associated with sperm size, though comparative studies have found both positive [e.g. 12], [ 31], [ 37], [ 38] and negative [e.g. 5], [ 12] associations, as well as no association [e.g. 12], [ 39], between these traits [reviewed in 23]. Thus, in contrast to studies of testes size and sperm numbers, the effects of sperm competition on sperm phenotype remain relatively unresolved. In particular, studies on sperm viability are almost entirely lacking; though at least in insects, sperm viability has been shown to influence competitive fertilization success [40], and polyandrous species have been shown to have a greater proportion of viable sperm available for ejaculates relative to monadrous species [41]. Consequently, further studies of sperm quality traits are needed to determine how sperm competition shapes inter-specific variation in sperm phenotype.

Distributed throughout New Guinea and Australia, the Maluridae are a family of passerine birds comprised of 27 species across five genera. In Australia, the malurid genera include the fairy-wrens (*Malurus*), emu-wrens (*Stipiturus*) and grasswrens (*Amytornis*) [42], [43]. Australian malurids are relatively small passerines (5 to 40 grams) and species tend to be similar in general morphology, life-history and ecology [42], [44]–[46]. Importantly, all species of Australian Maluridae are known or believed to be socially monogamous, with males and females forming long-term social pair bonds, but engage in extra-pair copulations [42].

The Australian malurids have become model system for the study of reproductive promiscuity because some species in this group show extremely high rates of extra-pair paternity (EPP; e.g. *M. cyaneus* [47]). In addition, the five species for which paternity data are available show remarkable variation in EPP rate, with between 5.8% and 95% of broods in a population containing one or more extra-pair offspring, and EPP accounting for as little as 4.4% or as much as 76% of all young in a population [46]–[52]. These differences suggest that species experience a broad range of sperm competition levels. Consistent with this idea, relative testis size is highly variable in this group, ranging from less than 1% to more than 6% of male body mass [46], [53]. In birds, the incidence of EPP varies across species from 0 to ∼76% of offspring (with values <5% considered to be unusual [54], [55]) and testes mass ranges from 0.01% to more than 9% of male body mass [11]. Thus the range of sperm competition levels observed in the Australian malurids reflects those observed across avian species generally, making them an ideal system for studying the evolutionary consequences of sperm competition for male reproductive biology.

In this study, we tested whether increasing levels of sperm competition were associated with variation in testis morphology and sperm quantity and quality using data from eight species of Australian Maluridae. Specifically, we used a phylogenetically-controlled, comparative approach to determine the relationship between sperm competition and the number of sperm in sperm reserves, the number of sperm in ejaculates, the proportion of motile sperm in ejaculates, the proportion of morphologically normal sperm and the proportion of viable sperm in sperm reserves, and the proportion of spermatogenic tissue, relative to interstitial tissue, contained within the testes. Importantly, all samples were collected and analyzed by a single individual specifically for this study. Thus, our study avoids some of the problems that confound other studies that rely on data gleaned from the literature, where collection methods may vary and inter-individual differences in measurement may lead to uncertain and sometimes erroneous results.

## Materials and Methods

### Ethical statement

All work was undertaken with approval from the University of Chicago Animal Care and Use Committee (ACUP#71453), the Department of Environment and Heritage (South Australia) Wildlife Ethics Committee (Project No. 13/2004; Scientific Permit Q24832; AW licence No. 142), the Director-General of New South Wales Department of Primary Industries Animal Care and Ethics Committee (Trim File No. 06/3846; NSW NPWS scientific licence S12048), James Cook University Animal Ethics Review Committee (approval #A1004), and the Environmental Protection Agency (EPA) of Queensland. Finally, export of samples from Australia was approved by the Australian Government Department of Environment and Heritage (WT2005-10120 and WT2006-10958).

### Study species and general field methods

Eight species of Australian Maluridae were studied over a three-year period (2004–2006). Species included the superb (*M. cyaneus cyanochlamys*), splendid (*M. splendens melanotus*), variegated (*M. lamberti assimilis*), blue-breasted (*M. pulcherrimus*), white-winged (*M. leucopterus leuconotus*), and red-backed fairy-wrens (*M. melanocephalus*), the southern emu-wren (*S. malachurus malachurus*), and the striated grasswren (*A. striatus striatus*). Populations were studied at several sites throughout southern and eastern Australia: superb fairy-wrens were studied at Murray River National Park, South Australia (140°32′E, 34°20′S); splendid, white-winged, and variegated fairy-wrens were studied at Brookfield Conservation Park, South Australia (139°29′E, 34°20′S); blue-breasted fairy-wrens were studied at Lincoln National Park, South Australia (135°52′E, 34°52′S); red-backed fairy-wrens were studied at Moomin Reservoir and Kalinvale Farm, near Herberton, Queensland (145°23′E, 17°23′S); southern emu-wrens were studied near Smith's Lake, New South Wales (152°28′E, 32°22′S); and striated grasswrens were studied at Pooginook and Cooltong Conservation Parks, near Berri. South Australia (140°35′E, 34°16′S).

Birds were captured in mist nets set on their home territory. Upon capture, birds were weighed to the nearest 0.1 g using a Pesola spring balance. Additionally, the length (L; measured from the anterior edge of the cloacal vent to the posterior edge of the protuberance, thus excluding the cloacal tip), width (W) and depth (D) of the cloacal protuberance was measured and cloacal protuberance volume was estimated as volume = π(D/2×W/2)×L [56]. All CP measurements were taken by one of us (MR) to minimize sampling error. We included data only from males in breeding condition, indicated by behavioral, morphological or physiological characteristics (e.g. courtship displays, breeding plumage, enlarged CP, active spermatogenesis).

### Testes size and morphology

We collected three male striated grasswrens, 17 male red-backed fairy-wrens and six males of each of the following species: superb, splendid, variegated, blue-breasted, and white-winged fairy-wrens and the southern emu-wren. We quantified fresh testes weight (wet mass) for both the left and right testis to the nearest 0.01 g using an electronic balance (Ohaus Navigator) and calculated the combined testes mass (CTM) as the sum of the left and right testis mass. We also calculated the gonadosomatic index (GSI), where GSI = (combined gonad weight/body weight)×100 [57]. Testes were then fixed in 10% neutral buffered formalin and transferred to 70% ethanol for transport and later histological work.

We examined the relative proportion of sperm producing tissue, compared to interstitial tissue, in testes using standard histological techniques and image analysis. Following fixation, we dehydrated and cleaned each testis via a series of increasing alcohol concentrations (70%, 80%, 95%, 100%) and two changes of xylene, and then passed the tissue through four changes of infiltration paraffin (paraffin type 1, Richard-Allan Scientific) at 60°C. We then embedded testes in paraffin (paraffin type 9, Richard-Allan Scientific) and cut 5-µm thick sections using a microtome (HM315, Microm) and stained sections with haematoxylin-eosin. We captured digital images of four non-sequential sections of each testis using a Leitz Laborlux S compound light microscope (at 20× magnification), Spot insight camera (model 14.2) and Spot for mac (version 4.1.1) image capture software (Diagnostic Instruments, Inc). For each section, we measured the proportion of seminiferous tubule tissue (relative to interstitial tissue) using Image-J software and calculated the proportion of tissue per testis by averaging the values from the four sections. The total proportion of sperm producing tissue in the testes of an individual was calculated by averaging the values from the left and right testis.

### Sperm quantity and quality

Sperm samples were collected using standard cloacal massage techniques [58]–[60]. Exuded semen was collected in 10 µl micro-capillary tubes, transferred to micro-centrifuge tubes containing a known volume of Lago Formulation Avian Semen Extender (Hygieia Biological Laboratories, Woodland, CA, USA), which is formulated to maintain sperm membrane integrity for a period of six hours or more, and mixed thoroughly. All ejaculate samples were collected and analyzed within three hours of collection using standard techniques by a single person (MR).

We measured sperm quantity as both as the number of sperm in ejaculates (i.e. sperm collected via cloacal massage) and the total number of sperm in the seminal glomera (i.e. sperm reserves). In the first instance, sperm density was determined in two aliquots of diluted semen using a calibrated Makler counting chamber (repeatability of counts within ejaculates: *r* = 0.93, *P*<0.0001; sensu [61]) and total sperm number was calculated by taking into account the sample dilution and the volume of semen recovered. Next, to quantify the number of sperm stored by each male, we first isolated the seminal glomera from males collected for testis size examination, measured the fresh weight (wet mass) of the left and right seminal glomerus to the nearest 0.01 g using an electronic balance (Ohaus Navigator), and calculated the combined seminal glomera mass as the sum of the left and right glomerus mass. Following this, sperm was flushed from each glomerus into a known volume of Lago Formulation Avian Semen Extender and sperm density, based on two aliquots (repeatability of counts within glomera: *r* = 0.95, *P*<0.0001; sensu [61]), and total count quantified using a Makler counting chamber. The total number of stored sperm was then calculated by summing the sperm count from the left and right glomerus and, when ejaculate samples were collected prior to dissection, the ejaculate sample sperm count. Finally, because CP size reflects sperm numbers [14], [55] and has been used as a proxy for sperm production in passerines [62], we include both CP volume and the mass of the seminal glomera as indirect measures of sperm quantity.

Measures of sperm quality were based on three sperm phenotypic traits. First, the proportion of motile sperm in ejaculates was estimated by visual examination [26], [63], [64]. Specifically, 10 µl of the diluted ejaculate was placed on a glass slide and the percentage of motile sperm (0–100%, in steps of 5%) assessed by examining several fields of view under phase contrast optics at 20 times magnification. Next, the viability and morphology of sperm from sperm reserves was assessed by examination of eosin-nigrosin stained sperm smears. Sperm viability was determined by recording the percentage of live (i.e. those excluding eosin) sperm [65]–[67], [ see also 68], [ 69]. For each male, we examined 100 sperm cells on each of two replicate smears (repeatability of viability counts: *r* = 0.83, *P*<0.0001; sensu [61]), for a total of 200 spermatozoa examined, and averaged these values to obtain a single metric of sperm viability. At the same time, sperm were categorized as either normal or abnormal (e.g. abnormal morphology of the head, midpiece or tail) to quantify the percentage of morphologically normal sperm in the sperm reserves of males [30], [59]. As for sperm viability, two replicate smears were examined and values averaged to provide a single measure of sperm morphology.

The measures we used were chosen instead of more elaborate assessment techniques (e.g. CASA) because many of our field sites lacked the necessary infrastructure for these methods. Furthermore, we chose metrics of sperm quality that appear to be less susceptible to change due to time since collection and temperature because many of our samples were collected from localities that prevented immediate assessment (i.e. birds were trapped long distances from roads and other locations were measures could be performed). For example, the percentage of motile sperm in ejaculates is reported to be stable for several hours after collection [26], [70], across a wide range of temperatures (e.g. 24–37°C, [71]).

### Statistical analysis

We collected a total of 349 ejaculate samples from eight species over three years. Males that did not produce an ejaculate sample were not included in the analysis and, because individual males were often sampled over consecutive years or more than once within a season, each male is included only once in the analysis (3–87 males per species, for details of sample size for all traits see Supplementary Table 1). However, as not all parameters were successfully sampled for all individuals, sample sizes vary slightly for the different metrics of sperm quantity and quality. Non-normal data distributions were normalized using log (ln)-transformations (CTM, body mass), and all proportion data (proportion of interstitial tissue, proportion of motile, viable and morphologically normal sperm) were arcsine-transformed prior to analysis. All statistics were performed using the R (2.12.0) software package [72].

Because comparative studies can be confounded by non-independence of data as a result of common ancestry [73], we used a generalized least-squares approach in a phylogenetic framework [74], [75] implemented in the APE package [76]. Additionally, we tested for phylogenetic dependence of traits by estimating the phylogenetic scaling parameter λ, where values of λ close to 0 indicate phylogenetic independence, while values close to 1 indicate phylogenetic dependence. Finally, likelihood-ratio testes were used to compare the model with the maximum likelihood value of λ differed from models with λ values of 0 or 1. We used a phylogeny based on allozyme data [77], which has been recently confirmed using DNA evidence [78]. The striated grasswren, which was not present on this tree, was replaced with its congener the black grasswren (*A. housei*). Because branch length information was unknown, we assumed a punctuated model of evolution (i.e. set all branch lengths equal) for all analyses. We estimated sperm competition level by including both CTM and male body mass (both log-transformed) as independent variables in the multiple regression models.

## Results

**Figure 1:**
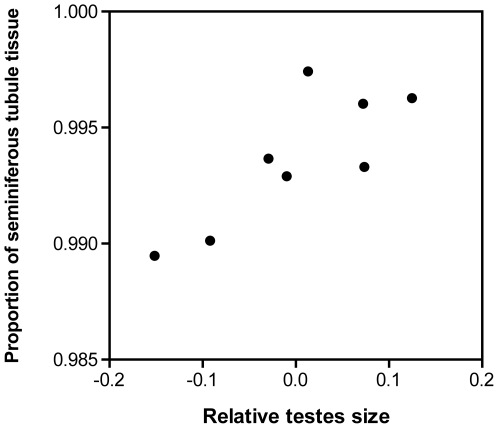
Relationship between relative testes size and the proportion of spermatogenic tissue contained within the testes in eight species of Australian Maluridae. Figure is not controlled for phylogeny (unlike analysis) and relative testes mass indicates the use of residual values from a linear regression of testis mass on body mass. Each data point represents a species. For further statistical details see main text.

Across the eight species of Maluridae, mean CTM ranged from 0.05 to 0.34 g, or, when expressed as the gonadosomatic index, from 0.62 to 4.45% of male body mass (Table 1). Combined testes mass was not related to male body mass (r^2^ = 0.21, *F* = 1.60, *P* = 0.25, λ = 0.99). The testes were predominately comprised of densely packed spermatogenic tissue, with interstitial tissue generally accounting for less than 1% (range 0.26 to 1.01%) of total testicular material. The proportion of spermatogenic tissue in the testes was positively associated with the level of sperm competition (Fig. 1). Specifically, using a GLS multiple regression corrected for phylogeny, we found a significant association between the proportion of seminiferous tubule tissue in the testes and both CTM (*t* = 3.39, *P* = 0.019) and body mass (*t* = −3.88, *P* = 0.012). The estimated λ value for this model was 0.999, and the model including the maximum-likelihood estimate of λ was not significantly different from either the model with λ set to 0 (*P* = 0.12) or the model with λ set to 1 (*P* = 1).

**Table 1:** Descriptive statistics of testis morphology and sperm quantity in eight species of Australian Maluridae.

	Superb Fairy-wren	Splendid Fairy-wren	Variegated Fairy-wren	Blue-breasted Fairy-wren	White-winged Fairy-wren	Red-backed Fairy-wren	Southern Emu-wren	Striated Grasswren
**Body mass (g)**	8.93±0.22 (6)	9.45±0.06 (94)	8.29±0.12 (33)	9.27±0.14 (15)	7.7±0.12 (13)	7.57±0.05 (76)	7.32±0.11 (6)	19.23±0.27 (3)
**CTM (g)**	0.29±0.04	0.30±0.02	0.18±0.01	0.13±0.02	0.33±0.02	0.21±0.01	0.045±0.004	0.34±0.009
**GSI**	3.39±0.48	3.56±0.25	2.38±0.15	1.49±0.24	4.45±0.23	2.95±0.18	0.62±0.06	1.81±0.04
**Sperm tissue**	99.60±0.11	99.33±0.09	99.37±0.05	99.01±0.25	99.63±0.06	99.74±0.06	98.99±0.12	99.29±0.03
**CP volume (mm^3^)**	105.08±11.8 (6)	96.99±3.27 (94)	36.44±3.15 (34)	31.39±3.36 (14)	86.96±8.63 (13)	128.24±3.36 (76)	0 (6)	94.15±9.47 (3)
**SG mass (g)**	0.09±0.007 (6)	0.09±0.01 (6)	0.05±0.006 (5)	0.04±0.01 (5)	0.09±0.006 (6)	0.05±0.004 (16)	0.01±0.002 (6)	0.05±0.01 (3)
**Sperm reserves (×10^6^)**	318.69±53.3 (6)	276.47±9.48 (6)	161.82±29.4 (5)	94.55±23.45 (5)	276.66±16.7 (6)	195.0±0.01 (16)	12.58±4.59 (6)	42.46±6.14 (3)
**Ejaculate size (×10^6^)**	33.36±8.96 (6)	48.37±3.82 (87)	8.54±1.64 (24)	15.16±2.49 (15)	45.69±5.15 (13)	36.13±3.0 (76)	0* (6)	6.56±2.18 (3)

CTM = combined testes mass, GSI = gonadosomatic index.

Cloacal protuberance (CP) volume and seminal glomera (SG) mass represent indirect measures of sperm quantity, while the number of sperm in sperm stores (sperm reserves) and the number of sperm in ejaculate samples (ejaculate size) represent direct measures of sperm quantity.

Species differed significantly in all four measures of sperm quantity: CP volume (ANOVA: *F*_7,238_ = 43.66, *P*<0.001), SG mass (ANOVA, *F*_7,45_ = 20.96, *P*<0.001), sperm reserves (ANOVA, *F*_7,46_ = 18.94, *P*<0.001), and ejaculate size (ANOVA, *F*_6,217_ = 8.87, *P*<0.001).

*Ejaculate samples were not collected from the southern emu-wren due to logistical problems during the 2006 field season.

Sample sizes for all traits are shown in Supplementary Table 1.

The four measures of sperm quantity differed significantly across the species (Table 1). After controlling for phylogeny, we found a significant association between sperm competition level and both indirect measures of sperm quantity: CP volume and seminal glomera mass. In a model including both CTM and body mass, both CP volume and the mass of the seminal glomera were independent of body mass, but covaried positively with CTM (Table 2a). Similarly, we found a positive association between the number of sperm in the seminal glomera (i.e. sperm reserves) and testes size, but not body size, of males (Table 2a). Finally, there was also a trend towards a positive association between the number of sperm in ejaculate samples and CTM across species, whereas there was no relationship between sperm numbers in the ejaculate and body mass of males (Table 2a).

**Table 2:** Multiple regression analyses controlling for phylogeny (GLS) of sperm quantity and quality in relation to combined testis mass and body mass across eight species of Australian Maluridae.

	predictor	slope	*t*	*P*	λ
**(a) Sperm quantity**					
CP volume	testis mass	58.37	2.89	0.03	<0.001 ^1.0; 0.09^
	body mass	−45.39	−1.34	0.24	
Seminal glomera mass	testis mass	0.04	4.32	0.007	<0.001 ^1.0; 0.04^
	body mass	−0.03	−1.94	0.11	
Sperm stores	testis mass	152.33	3.14	0.02	<0.001 ^1.0; 0.07^
	body mass	−172.23	−2.11	0.09	
Ejaculate sperm count	testis mass	31.05	2.44	0.07	<0.001 ^1.0; 0.04^
	body mass	−40.69	−2.23	0.09	
**(b) Sperm quality**					
Motile sperm in ejaculates	testis mass	0.24	2.94	0.04	<0.001 ^1.0; 0.02^
	body mass	0.05	0.45	0.67	
Viable sperm in sperm reserves	testis mass	0.15	2.99	0.03	<0.001 ^1.0; 0.007^
	body mass	−0.04	−0.47	0.66	
Morphologically normal sperm in sperm reserves	testis mass	0.18	4.23	0.008	<0.001 ^0.14; 0.5^
	body mass	−0.36	−4.95	0.004	

The model including the maximum-likelihood value of λ was compared against the models including λ = 0 and λ = 1, and superscripts following the λ estimates indicate significance levels of the likelihood-ratio testes (first position: against λ = 0; second position: against λ = 1).

**Figure 2:**
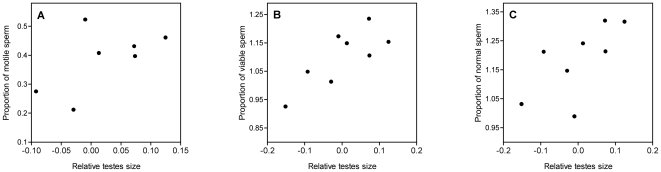
Relationship between the level of sperm competition (measured as relative testes size) and a) the proportion of motile sperm in ejaculates, b) the proportion of viable sperm in sperm stores, and c) the proportion of morphologically normal sperm in sperm stores. Unlike all analyses, figures are not controlled for phylogeny. The values for relative testes mass are the residuals obtained from a linear regression of testes mass on body mass. Proportion data are arcsine-transformed. Each data point represents a species. See main text for further statistical details.

As for sperm quantity, measures of sperm quality varied significantly across species (ANOVA: motility: *F*_6,211_ = 3.42, *P* = 0.003; viability: *F*_7,217_ = 4.96, *P*<0.001; morphology: *F*_7,218_ = 6.75, *P*<0.001). We found a positive relationship between the percent of motile sperm in an ejaculate and the level of sperm competition as measured by relative testes size (CTM with body mass as a covariate; Table 2b, Fig. 2a). Additionally, both the percent of viable sperm and the percent of morphologically normal sperm in sperm stores increased with increasing relative testes size (Table 2b, Fig. 2b, 2c). Neither the proportion of motile sperm or the proportion of viable sperm were associated with body mass, but the proportion of morphologically normal sperm was negatively correlated with male body mass (Table 2b). We obtained similar result in all analyses using raw species values (i.e. no phylogenetic control; see Supplementary Table 2).

## Discussion

For males, a general evolutionary response to sperm competition is an increase in testes size [2]–[7], such that relative testes mass is a common measure of sperm competition in many taxa, including birds [e.g. 9]–[12]. In the current study, we found a more than seven-fold variation in relative testis size across species of Australian Maluridae, which suggests that these species experience a broad range of sperm competition levels: from low in the southern emu-wren, low to intermediate in the striated grasswren and some species of fairy-wrens, to high in other species of fairy-wren. Our results also revealed a positive association between the level of sperm competition and both the quantity and quality of sperm. Furthermore, we found that increasing levels of sperm competition were associated with a relatively greater proportion of seminiferous tubule tissue in the testes. Thus, across species of Australian Maluridae, post-copulatory sexual selection appears to select for a suite of male traits that may influence competitive fertilization success.

Under conditions of sperm competition, male fertilization success is commonly determined by the number of sperm, relative to rival males, transferred during copulation [62], [79]–[81]. We showed that across species the number of sperm in both sperm reserves and in ejaculates was positively associated with the level of sperm competition. Generally, large sperm reserves are thought to secure paternity success via frequent copulation (i.e. few sperm per copulation/many copulations; [14], [82]). In the fairy-wrens, however, copulation is believed to be relatively infrequent and under female control [41], [83], [84]. Furthermore, in some fairy-wrens (i.e. splendid, superb, white-winged and red-backed fairy-wrens), the number of sperm in ejaculates is very large (*ca.* 33–48×10^6^ sperm) compared with data available for a few other species. For example, in the zebra finch (*Taeniopygia guttata*), the number of sperm in ejaculates ranged from 0.17–5.29×10^6^ [85]; and in the much larger Japanese quail (*Coturnix coturnix*; mass ∼120 g), the mean number of sperm in ejaculates was 12×10^6^ [86]. Even in the promiscuous red-winged blackbird (*Agelaius phoeniceus*), sperm numbers do not match those observed in fairy-wrens (mean sperm number: 12.5×10^6^ [87]). The large sperm reserves and ejaculate size of fairy-wrens may therefore represent an alternate paternity strategy, whereby males maximize paternity success via the transfer of large numbers of sperm in one or a few ejaculates (i.e. many sperm per copulation/few copulations).

While the fitness benefits of increased sperm quantity are well understood, the mechanisms of sperm production and the potential selective forces operating on these mechanisms remain relatively unexplored. High sperm production can be achieved via an increase in relative testis size, an increase in the efficiency of sperm production (daily sperm production per unit of testes tissue, [16], [88]), or a combination of these factors. Sperm production efficiency may be increased if the proportion of spermatogenic tissue (relative to interstitial tissue) in the testes increases. In the current study, we demonstrated that increasing levels of sperm competition are accompanied by an increase in the relative proportion of seminiferous tubule tissue in the testes of Australian malurid species, which is consistent with recent results from two other avian families [22]. The congruent results of these two studies suggests that selection for sperm production rates via an increase in the amount of sperm-producing tissue may be a common evolutionary response to sperm competition, at least in passerine birds. However, the amount of spermatogenic tissue in the testes varies over a very small range in the Maluridae, and whether these small differences translate into significant differences in sperm production is unknown. An alternative, and perhaps equally likely, explanation of these results is that a correlation between testis size and spermatogenic tissue arises as an artefact of testicular structure scaling: if interstitial tissue does not scale isometrically with testicular size, larger testes will show a proportionally greater amount of sperm-producing tissue simply because the proportion of interstitial tissue decreases. These hypotheses are not necessarily mutually exclusive, however, and it is clear that additional studies are needed in order to understand the factors shaping testicular morphology.

The efficiency of sperm production may also be influenced by the duration of the cycle of the seminiferous epithelium (and consequently the rate of spermatogenesis). As Lüpold and coworkers [22] suggest, data regarding spermatogenesis in birds is limited in scope and focused on a few domesticated species (e.g. japanese quail [86], [89], turkey [90]). In mammals however, elevated levels of sperm competition are associated with a shorter seminiferous epithelium cycle length [91]–[93]. These studies highlight the need to integrate data on relative testis size and the kinetics of spermatogenesis in order to understand selection on sperm production in males. Finally, as sperm production may also be shaped by the number of mitotic divisions of spermatogonia, the capacity of sperm to survive and complete spermatogenesis, and the duration of sperm transport, future studies should also aim to investigate these aspects of spermatogenesis.

In addition to selection for more sperm, postcopulatory sexual selection appears to select for higher quality sperm in the Maluridae. In birds, fertilization success is determined by sperm mobility [25], [94] and the percentage of motile sperm in an ejaculate [26]. Thus, selection at the intraspecific level appears to translate to macroevolutionary patterns of increased swimming speed [31] and a greater percentage of motile sperm (this study) with increasing levels of sperm competition. There are two main reasons to assume that the percent of viable sperm and morphologically normal sperm may also influence fertilization success in birds. First, only viable and morphologically normal sperm enter the sperm storage tubules of females [95], [96], and second, morphologically abnormal sperm appear less effective at reaching the infundibulum (the site of fertilization) and penetrating an egg to achieve fertilization [69]. In addition, these traits have been shown to influence fertility in other taxa (e.g. sperm viability, insects, [40]; morphologically normal sperm, deer, [30]). We found significant interspecific variation in the proportion of viable and morphologically normal sperm. Specifically, our results showed that species experiencing higher levels of sperm competition had a greater proportion of morphologically normal and viable sperm available for copulation, suggesting sperm viability and morphology are favored under conditions of sperm competition.

The occurrence of dead or morphologically abnormal sperm is generally attributed to production errors during spermatogenesis [97], [98], or may result from increased replication-dependent mutations associated with increased sperm production [99] if these mutations alter sperm phenotype. Our results suggest that postcopulatory sexual selection may favor mechanisms that minimize sperm production errors and maintain sperm integrity during transport and storage. In the testes, oxidative stress results in a reduced capacity to differentiate normal sperm [100]. Additionally, oxidative stress reduces both sperm motility and viability [101], [102]. Consequently, selection may target mechanisms underlying sperm function aimed at avoiding oxidative stress and preventing oxidative damage to sperm structures (see [103]). Alternatively, males may be able to allocate substances to their testes and semen that protect sperm integrity and influence sperm quality (see [104], [105]); as has been observed in *Drosophila* (e.g. protease inhibitors, anti-microbial peptides [106]).

Regardless of the underlying cause(s) of extra-pair copulation, female promiscuity has significant evolutionary consequences for the reproductive biology of male Australian malurids. In the current study, we show that postcopulatory sexual selection is associated with an increase in the quantity and quality of sperm in sperm reserves and ejaculates. Furthermore, we demonstrate that increased sperm quantity is likely achieved, not only through an increase in testes size, but through selection for greater sperm production via an increase in the relative amount of sperm-producing tissue contained within the testes. Future intraspecific studies that investigate the relative importance of these sperm traits on male paternity success will provide a more comprehensive understanding of how these macroevolutionary patterns in sperm traits have evolved.

## Supporting Information

Table S1 **Number of males contributing to the analysis of male reproductive traits (testicular morphology, sperm quantity and sperm quality) for all fairy-wren (F-W), emu-wren and grasswren species.**
(DOC)

Table S2 **Results of all analyses using raw species values (i.e. λ set to 0 in GLS analysis, no phylogenetic control).**
(DOC)
