# Supplementary material for: Sperm Competition Selects for Sperm Quantity and Quality in the Australian Maluridae
Source: PLoS One. 2011 Jan 25;6(1):e15720. doi: 10.1371/journal.pone.0015720 (PMC3026798; doi:10.1371/journal.pone.0015720)
Supplement: Table S1 — Number of males contributing to the analysis of male reproductive traits (testicular morphology, sperm quantity and sperm quality) for all fairy-wren (F-W), emu-wren and grasswren species. (DOC) [file pone.0015720.s001.doc]

**Supplementary Table 1. Number of males contributing to the analysis of male reproductive traits (testicular morphology, sperm quantity and sperm quality) for all fairy-wren (F-W), emu-wren and grasswren species.**

|  | **Superb**  **F-W** | **Splendid F-W** † | **Variegated F-W** † | **Blue-breasted F-W** | **White-winged F-W** † | **Red-backed F-W** † | **Southern Emu-wren** | **Striated Grasswren** |
| --- | --- | --- | --- | --- | --- | --- | --- | --- |
| **Body mass** | 6 | 87 | 33 | 15 | 13 | 76 | 6 | 3 |
| **Testicular traits** | 6 | 6 | 5 | 5 | 6 | 16 | 6 | 3 |
| **CP volume** | 6 | 87 | 34 | 14 | 13 | 76 | 6 | 3 |
| **SG mass** | 6 | 6 | 5 | 5 | 6 | 16 | 6 | 3 |
| **Sperm reserves** | 6 | 6 | 5 | 5 | 6 | 16 | 6 | 3 |
| **Ejaculate sperm** | 6 | 87 | 24 | 15 | 13 | 76 | 0* | 3 |
| **Sperm motility** | 6 | 86 | 21 | 15 | 12 | 75 | 0* | 3 |
| **Sperm viability** | 6 | 87 | 21 | 15 | 13 | 76 | 4 | 3 |
| **Sperm morphology** | 6 | 87 | 22 | 15 | 13 | 76 | 4 | 3 |

Body mass is for males only.

Testicular traits include combined testes mass (CTM), gonadosomatic index (GSI), and proportion of spermatogenic tissue.

* Ejaculate samples were not collected from the southern emu-wren due to logistical problems during the 2006 field season.

† Samples were collected over multiple years, but all samples were collected during the peak of the breeding season during each sampling year.
