# Supplementary material for: Sperm Competition Selects for Sperm Quantity and Quality in the Australian Maluridae
Source: PLoS One. 2011 Jan 25;6(1):e15720. doi: 10.1371/journal.pone.0015720 (PMC3026798; doi:10.1371/journal.pone.0015720)
Supplement: Table S2 — Results of all analyses using raw species values (i.e. λ set to 0 in GLS analysis, no phylogenetic control). (DOC) [file pone.0015720.s002.doc]

**Supplementary Table 2a-c. Results of all analyses using raw species values (i.e. λ set to 0 in GLS analysis, no phylogenetic control).**

|  | **predictor** | **slope** | ***t*** | ***P*** |
| --- | --- | --- | --- | --- |
| **(a) Testis morphology** |  |  |  |  |
| Combined testes mass | body mass | 1.37 | 1.32 | 0.24 |
| Sperm-producing tissue | testis mass | 0.03 | 3.64 | 0.02 |
|  | body mass | -0.04 | -1.95 | 0.11* |
| **(b) Sperm quantity** |  |  |  |  |
| CP volume | testis mass | 57.44 | 3.96 | 0.01 |
|  | body mass | -26.38 | -0.63 | 0.56 |
| Seminal glomera mass | testis mass | 0.04 | 6.48 | 0.001 |
|  | body mass | -0.05 | -2.38 | 0.06 |
| Sperm stores | testis mass | 149.29 | 4.39 | 0.007 |
|  | body mass | -243.79 | -2.47 | 0.06 |
| Ejaculate sperm count | testis mass | 35.75 | 2.54 | 0.06 |
|  | body mass | -45.52 | -3.70 | 0.02* |
| **(c) Sperm quality** |  |  |  |  |
| Motile sperm in ejaculates | testis mass | 0.24 | 2.82 | 0.04 |
|  | body mass | 0.07 | 0.92 | 0.41 |
| Viable sperm in sperm reserves | testis mass | 0.12 | 4.55 | 0.006 |
|  | body mass | -0.002 | -0.03 | 0.98 |
| Morphologically normal sperm in sperm reserves | testis mass | 0.14 | 3.27 | 0.02 |
|  | body mass | -0.31 | -2.61 | 0.047 |

* Indicates different result to those found in analyses controlling for phylogeny.
